# Resistance training suppresses accumulation of senescent fibro-adipogenic progenitors and senescence-associated secretory phenotype in aging rat skeletal muscle

**DOI:** 10.1007/s11357-024-01338-2

**Published:** 2024-09-19

**Authors:** Yung-Li Hung, Ayami Sato, Yuka Takino, Akihito Ishigami, Shuichi Machida

**Affiliations:** 1 Japan Society for the Promotion of Science, 5-3-1 Kojimachi, Chiyoda-Ku, Tokyo, 102-0083 Japan; 2 Graduate School of Health and Sports Science, Juntendo University, 1-1 Hirakagakuendai, Inzai, Chiba 270-1695 Japan; 3 Molecular Regulation of Aging, Tokyo Metropolitan Institute for Geriatrics and Gerontology, 35-2 Sakae-Cho, Itabashi-Ku, Tokyo, 173-0015 Japan; 4 Institute of Health and Sports Science & Medicine, Juntendo University, 1-1 Hirakagakuendai, Inzai, Chiba 270-1695 Japan

**Keywords:** Sarcopenia, Senescent cells, Resistance training, Fibro-adipogenic progenitors

## Abstract

Accumulation of senescent cells in tissues contributes to multiple aging-related pathologies. Senescent fibro-adipogenic progenitors (FAPs) contribute to aging-related muscle atrophy. Resistance training can help to maintain skeletal muscle mass, improve mobility, and reduce certain health risks commonly associated with aging. We investigated, using rat model, the impact of resistance training on FAPs in aging skeletal muscle, which remains unclear. Twenty-two-month-old female rats were divided into sedentary and training groups. The training group rodents were trained to climb a ladder while bearing a load for 20 training sessions over 2 months, after which, the flexor hallucis longus muscles were collected and analyzed. Senescent cells were identified using a senescence-associated β-galactosidase stain and p21 immunohistochemistry (IHC), and FAPs were identified using platelet-derived growth factor receptor alpha IHC. The results indicate that resistance training in rats prevented aging-associated skeletal muscle atrophy and suppressed M2 polarization of macrophages. The number of senescent cells was significantly reduced in the 24-month-old training group, with most of them being FAPs. Conversely, the number of senescent FAPs increased significantly in the 24-month-old sedentary group compared with that in the 18-month-old sedentary group. The number of senescent FAPs in the 24-month-old training group decreased significantly. Resistance training also suppressed the senescence-associated secretory phenotype (SASP). The killer T cell-specific marker, CD8α, was elevated in the skeletal muscles of the aging rats following resistance training, indicating upregulation of recognition and elimination of senescent cells. Overall, resistance training suppressed the accumulation of senescent FAPs and acquisition of SASP in aging skeletal muscles.

**Supplementary Information:**

The online version contains supplementary material available at 10.1007/s11357-024-01338-2.

## Introduction

Cellular senescence causes permanent cell cycle arrest in response to various intrinsic and extrinsic stimuli. It is a defense mechanism that limits the proliferation of damaged cells, removes harmful factors, and prevents malignant cell transformation. Cell cycle arrest is initiated by activation of cyclin-dependent kinase inhibitor p21 (Cdkn1a)- and p16 (Cdkn2a)-mediated tumor suppressor pathways. Senescent cells are characterized by increased expression of p16 and p21, increased activity of senescence-associated β-galactosidase (SA-β-gal), as well as histological changes [1]. The senescent cells secrete proinflammatory cytokines which are referred to as the senescence-associated secretory phenotype (SASP), to recruit immune cells to clear senescent cells [2]. The aging-related accumulation of senescent cells is associated with decline in senescent cell clearance by the deteriorating immune system [3, 4]. The accumulation of senescent cells in tissues contributes to multiple aging-related pathologies [5]. Pharmacological clearance of senescent cells, or senolytic treatment, can delay the onset of aging-related diseases by selectively eliminating senescent cells [6–8]. Sarcopenia is a common age-related disorder in elderly adults that results in a gradual loss of skeletal muscle mass and strength. It causes functional deterioration and increases the risk of falls, frailty, and mortality [9]. The age-related senescent cell accumulation and inflammation (inflammaging) are involved in the pathophysiological mechanisms of sarcopenia, contributing to its development [10]. Reducing the accumulation senescent cells in skeletal muscles can promote aging skeletal muscle health [11] and improve sarcopenia [12].

Skeletal muscle is composed of terminally differentiated multi-nucleated muscle cells called muscle fibers, mitotic mononuclear cells called fibro-adipogenic progenitors (FAPs), endothelial cells, satellite cells, and immune cells. [13]. The FAPs, particularly, play a critical role in homeostasis of skeletal muscle such as muscle regeneration and maintenance of skeletal muscle mass [14]. Senescent FAPs impair muscle satellite cell differentiation and contribute to skeletal muscle atrophy and fibrosis [15]. Elimination of senescent FAPs was shown to decrease SASP and improve muscle fiber size in progeria-aged skeletal muscles [16]. Thus, elimination of senescent FAPs may mitigate aging phenotype in skeletal muscles.

Several studies have demonstrated that exercise training can suppress age-related senescent cell accumulation in various tissues [17], and presumably exerts senolytic effects. Following 4 weeks of treadmill exercise, p21 protein expression was found to decrease in skeletal muscle in 19-month-old mice [18]. Moreover, resistance training improves skeletal muscle mass in aging [19]. The number of senescent cells was found to decrease in adipose tissue of older women following resistance training intervention [20]. However, the effects of resistance training on FAPs in aging skeletal muscle is unclear. Therefore, the present study aimed to investigate the effects of resistance training on FAPs in aging skeletal muscles. Using a rodent model, the senescent cell accumulation, senescent cell type, SASP, and immune cell markers in aging skeletal muscles, following resistance training, were evaluated. We hypothesized that resistance training suppresses accumulation of senescent FAPs in aging skeletal muscles.

## Methods

### Experimental animals

Seven 16-month-old, fifteen 22-month-old, and five 28–32-month-old female F344 rats were obtained from Charles River Laboratories Japan, Inc. (Yokohama, Japan), and housed in controlled environmental conditions (23 ± 1 °C, 55 ± 5% relative humidity) under a 12-h light/dark cycle with ad libitum access to water and standard laboratory diet. After subjecting the animals to 2 months of either climbing training or no exercise, the animals were categorized as follows: The 22-month-old rats were randomly assigned to either a sedentary group (24 mo-Sed, *n* = 7) or a climbing training group (24 mo-CT, *n* = 8), and the 16-month-old animals were categorized into a sedentary group (18 mo-Sed, *n* = 7). All animal experiments were conducted in compliance with the ethics approval from the Committee for Animal Experiment at Sakura Campus, Juntendo University (S3).

### Ladder climbing training

The climbing training procedure was adapted from a previously published protocol by our laboratory [21]. The climbing training group rats were trained to climb a 1-m-long ladder with rungs that were 2-cm apart and inclined at 85°; for one training session per 3 days, a total 20 training sessions for 2 months. Before the first 3-day training session, the rats were acclimated to ladder climbing without any load for 3 days. The starting carrying loads were 50, 75, 90, and 100% of body weight, with an additional carrying load of 30 g for each climb repetition up to 10 repetitions in the first training session. The load was progressively reduced when rats could climb 10 repetitions owing to the heavy load. This training session’s maximal carrying capacity was determined by the heaviest load carried by the rat, who successfully climbed to the top of the ladder. From the 2nd to 20th training session, the initial carrying loads were 50, 75, 90, and 100% of the previous training session’s maximum carrying capacity, and the load was gradually increased by 30 g.

### Muscle tissue sampling

After 48 h of the last training session, the flexor hallucis longus (FHL) muscles from both legs were carefully dissected, weighed, and frozen in liquid nitrogen for biochemical analysis or in isopentane cooled by liquid nitrogen for immunohistochemical analysis. Muscle tissue samples were stored at − 80 °C until analysis.

### Immunohistochemistry

Myosin heavy chain (MHC) typing of the midbelly region of the FHL muscles was used to determine the cross-sectional area (CSA) and analyze individual muscle fiber type. Frozen cross-Sects. (10 μm) were stained using mouse monoclonal antibodies against MHC I, IIa, IIx and rabbit polyclonal antibodies against laminin. Briefly, muscle sections were fixed in 4% paraformaldehyde/phosphate-buffered saline (PBS) on ice for 3 min. The sections were blocked in 10% normal goat serum (NGS)/PBS with 1% Triton X-100/PBS at 25 °C for 1 h, and subsequently incubated with primary antibodies at room temperature for 1 h. The primary antibodies, anti-MHC I antibodies (clone BA-F8; 1:100), anti-MHC IIa antibodies (clone SC-71; 1:100), anti-MHC IIx antibodies (6H-1; 1:100) (Developmental Studies Hybridoma Bank, Iowa, IA, USA), and anti-laminin antibodies (L9393; 1:100)(Sigma Aldrich, St. Louis, MO, USA), were diluted in 5% NGS/PBS.

For visualization of p21 and FAPs, the sections were fixed in 4% paraformaldehyde/PBS at 25 °C for 15 min. The sections were blocked in 10% NGS/PBS with 1% Triton X-100/PBS at 25 °C for 1 h, and were incubated with primary antibodies at 4 °C for overnight. The primary antibodies anti-p21 (556430; 1:100)(BD Biosciences, Franklin Lakes, NJ, USA) and anti-platelet-derived growth factor receptor (PDGFR)-α antibodies (ab203491; 1:100)(Abcam, Cambridge, UK) were diluted in 5% NGS/PBS.

For detection of p21 and macrophages, the sections were fixed in methanol at 25 °C for 10 min. The sections were blocked in 10% NGS/PBS with 1% Triton X-100/PBS at 25 °C for 1 h, and were incubated with primary antibodies at 4 °C for overnight. The primary antibodies anti-p21 (ab109199; 1:100)(Abcam), anti-CD68 (ab31630; 1:50)(Abcam), and anti-CD163 (MCA342R; 1:100) (Bio-Rad Laboratories, Hercules, CA, USA) were diluted in 5% NGS/PBS.

After incubation with primary antibody, the sections were washed with PBS and incubated for 1 h at 25 °C with secondary antibodies (goat anti-mouse IgG [Invitrogen, Carlsbad, CA, USA] for BA-F8, p21 (BD), CD68 and CD163, goat anti-mouse IgM [Invitrogen] for 6H-1, and goat anti-rabbit IgG [Invitrogen] for PDGFR-α, p21 (Abcam) and laminin) or wheat germ agglutinin (WGA) with Alexa Fluor (Invitrogen). The secondary antibodies were diluted 1:500 in 5% NGS/PBS. Images of the stained sections were observed under a fluorescence microscope (BZ-X800; Keyence, Osaka, Japan). CSA of approximately 200 fibers/muscle section and three muscle section/muscle was calculated using image analysis software Keyence analyzer (Keyence). For analysis of muscle fiber type composition, the number of muscle fibers was counted for each muscle fiber type from a total of approximately 500–600 fibers. For cell count analysis, the regions of interest were evaluated for 5.7 × 10^5^ μm^2^ per section and three sections per muscle.

### SA β-Gal staining

The SA β-Gal staining protocol for skeletal muscle was modified from Cazin et al. [22]. The frozen cross sections were fixed in 1% paraformaldehyde and 0.2% glutaraldehyde (FUJIFILM Wako Pure Chemicals, Osaka, Japan)/PBS at 25 °C for 4 min. After washing with PBS two times for 10 min, the sections were incubated in PBS (pH 5.5) for 30 min, followed by incubation of the sections in the 5-bromo-4-chloro-3-indolyl-β-D-galactopyranoside (X-gal) solution containing (4 mM K_3_Fe (CN)_6_, 4 mM K_4_Fe (CN)_6_, 2 mM MgCl_2_, and 400 μg/mL X-Gal in PBS, pH 5.5) (FUJIFILM Wako Pure Chemicals) in the dark at 37 °C for 72 h, with fresh X-gal solution added every 24 h. After incubation, the slides were washed with PBS three times for 10 min at 25 °C, and fixed in 1% paraformaldehyde/PBS for 30 min. After washing with PBS three times for 10 min, the slides were mounted with aqueous mounting medium (Vector Laboratories, Inc. Mowry Ave Newark, CA, USA).

### RNA isolation

The FHL muscle powder was homogenized in 1 mL ISOGEN (FUJIFILM Wako Pure Chemical), chloroform was added to the homogenate and shaken vigorously for 15 s and kept at 25 °C for 3 min. The mixture was then centrifuged at 15,000 × *g* for 15 min at 4 °C, and the aqueous phase was transferred to a fresh tube. Isopropanol was added to this aqueous solution to precipitate the RNA. After standing at 25 °C for 10 min, the tube was centrifuged at 15,000 × *g* for 15 min at 4 °C. The recovered RNA pellet was washed with 70% ethanol and subsequently centrifuged at 15,000 × *g* for 10 min at 4 °C. The RNA pellet was air-dried for 5 min, and the RNA was dissolved in RNase-free water. RNA extracts were treated with DNase (Invitrogen) to remove any residual genomic DNA. RNA concentrations were determined by measuring absorbance at 260 nm.

### Quantitative polymerase chain reaction (q-PCR)

cDNA was synthesized from RNA using SuperScript III reverse transcriptase (18080–044; Invitrogen) according to the manufacturer’s instructions. Real-time PCR was performed using the QuantStudio™ 3 System (Applied Biosystems, Foster City, CA, USA) and THUNDERBIRD SYBR® qPCR Mix (QPS201; Toyobo, Osaka, Japan) according to the manufacturer’s instructions. The amplification protocol consisted of denaturation at 95 °C for 10 min, followed by 40 cycles of 95 °C for 15 s and 60 °C for 1 min. A standard curve was plotted to quantitatively analyze mRNA expression levels; an aliquot of each experimental sample was used to generate the standard curve. The following primers were used: p21 forward 5′-CCGAGAACGGTGGAACTTTGAC-3′ and reverse 5′-GAACACGCTCCCAGACGTAGTTG-3′; tumor necrosis factor (TNF)-α forward 5′-AACACACGAGACGCTGAAGT-3′ and reverse 5′-TCCAGTGAGTTCCGAAAGCC-3′; interleukin (IL)-β forward 5′-TGACTTCACCATGGAACCCG-3′ and reverse 5′-GACCTGACTTGGCAGAGGAC-3′; IL-6 forward 5′-GGTTTGCCGAGTAGACCTCA-3′ and reverse 5′-GTGGCTAAGGACCAAGACCA-3′; and glyceraldehyde-3-phosphate dehydrogenase (GAPDH) forward 5′-TGAACGGGAAGCTCACTGG-3′ and reverse 5′- TCCACCACCCTGTTGCTGTA-3′. GAPDH was used for normalization.

### Western blotting

The muscle protein extracts (20 μg) were separated by electrophoresis using 12% sodium dodecyl sulfate polyacrylamide gels and electro-blotted onto polyvinylidene fluoride membranes. The blots were stained with Ponceau S to verify the equal loading of proteins. The membranes were blocked with 5% skim milk in Tris-buffered saline/Tween 20 (TBST) for 1 h at 25 °C and then incubated with anti-CD8α primary antibody (sc-7970; Santa Cruz Biotechnology. Inc, Dallas, TX, USA) at 4 °C overnight. The blots were washed three times with TBST and incubated with a 1:5000 dilution of horseradish peroxidase-linked secondary antibody (anti-mouse IgG; NA931; GE Healthcare UK Limited, Buckinghamshire, UK) for 1 h at 25 °C. After washing the blots three times with TBST, it was developed using an enhanced chemiluminescence substrate (Amersham, Buckinghamshire, UK). Band intensities were quantified using Image Lab v.5.2.1 software (Bio-Rad Laboratories).

### Statistical analyses

All data are expressed as means ± standard deviation (SD). Statistical analysis was performed via one-way analysis of variance (ANOVA) followed by the Tukey post hoc test or Fisher’s LSD test, two-way ANOVA followed by the Tukey post hoc test and unpaired Student’s *t*-tests using GraphPad Prism 5 software (San Diego, CA, USA). The comparison of muscle mass, muscle fiber CSA, p21^+^ cells, PDGFR-α^+^ cells (FAPs), CD68^+^ cells (pan macrophages), CD163^+^ cells (M2 macrophages), and SAPS among all groups was evaluated using one-way ANOVA followed by the Tukey post hoc test. The comparison of CD8α among all groups was evaluated using one-way ANOVA followed by the Fisher’s LSD test. The significant difference was set at ^*^p < 0.05 by Tukey post hoc test or ^*#*^*p* < 0.05 by Fisher’s LSD test. The comparison of body weight among all groups was evaluated using two-way ANOVA followed by the Tukey post hoc test. The comparison of SA β-Gal^+^ cells among all groups was evaluated using one-way ANOVA followed by the Tukey post hoc test, and that between 24 mo-Sed and 24 mo-CT was evaluated using unpaired Student’s *t*-tests. The significant difference was set at ^+^*p* < 0.05 by unpaired Student’s *t*-tests that were used to evaluate differences between the groups with respect to climbing training.

## Results

### Resistance training prevented aging-associated skeletal muscle atrophy

**Fig. 1:**
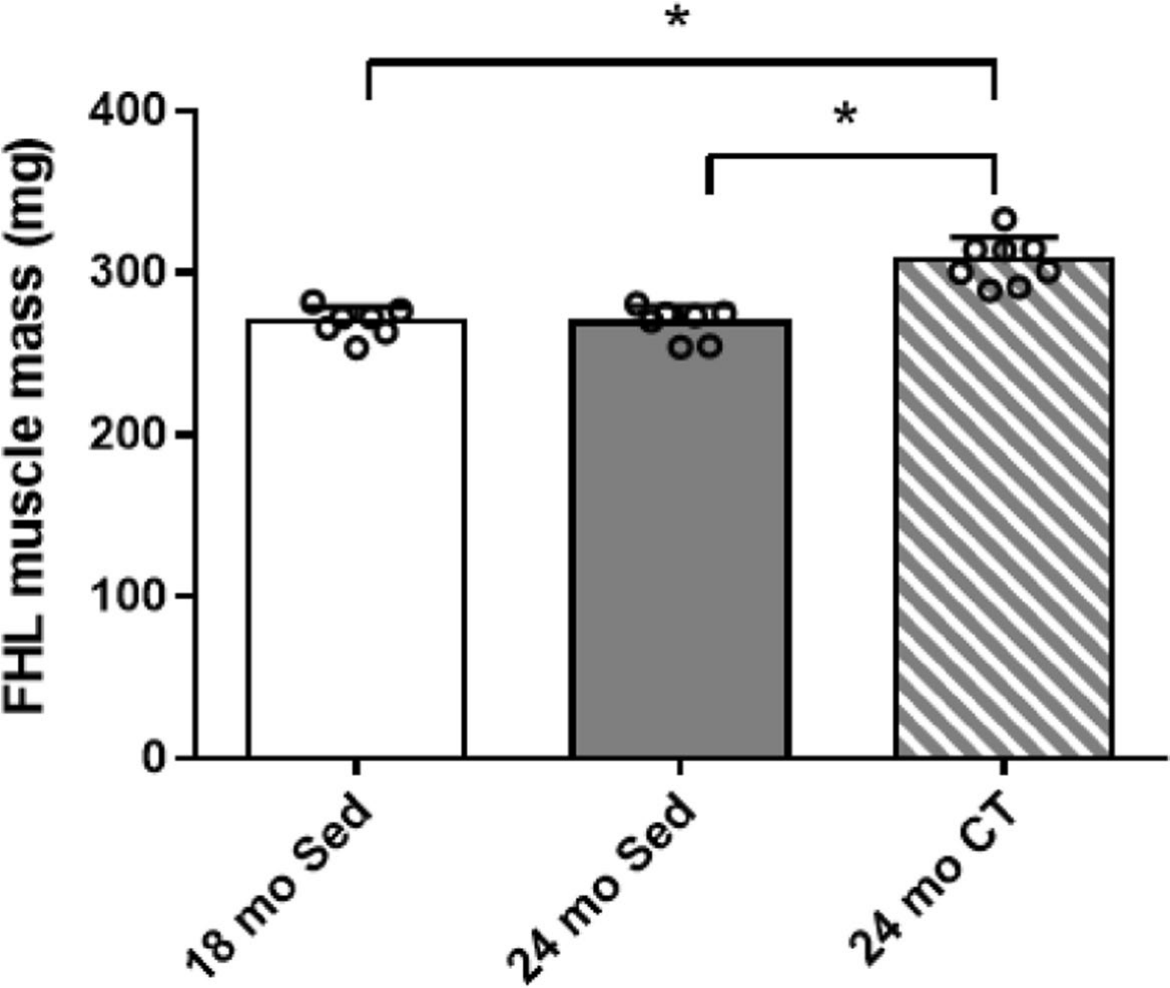
Effects of climbing training on the muscle mass of flexor hallucis longus (FHL) muscle. Values are presented as the mean ± SD (*n* = 7–8); *: *p* < 0.05, Tukey post hoc test, 18 mo Sed, 18-month-old sedentary; 24 mo Sed, 24-month-old sedentary; 24 mo CT, 24-month-old climbing training; SD, standard deviation

**Fig. 2:**
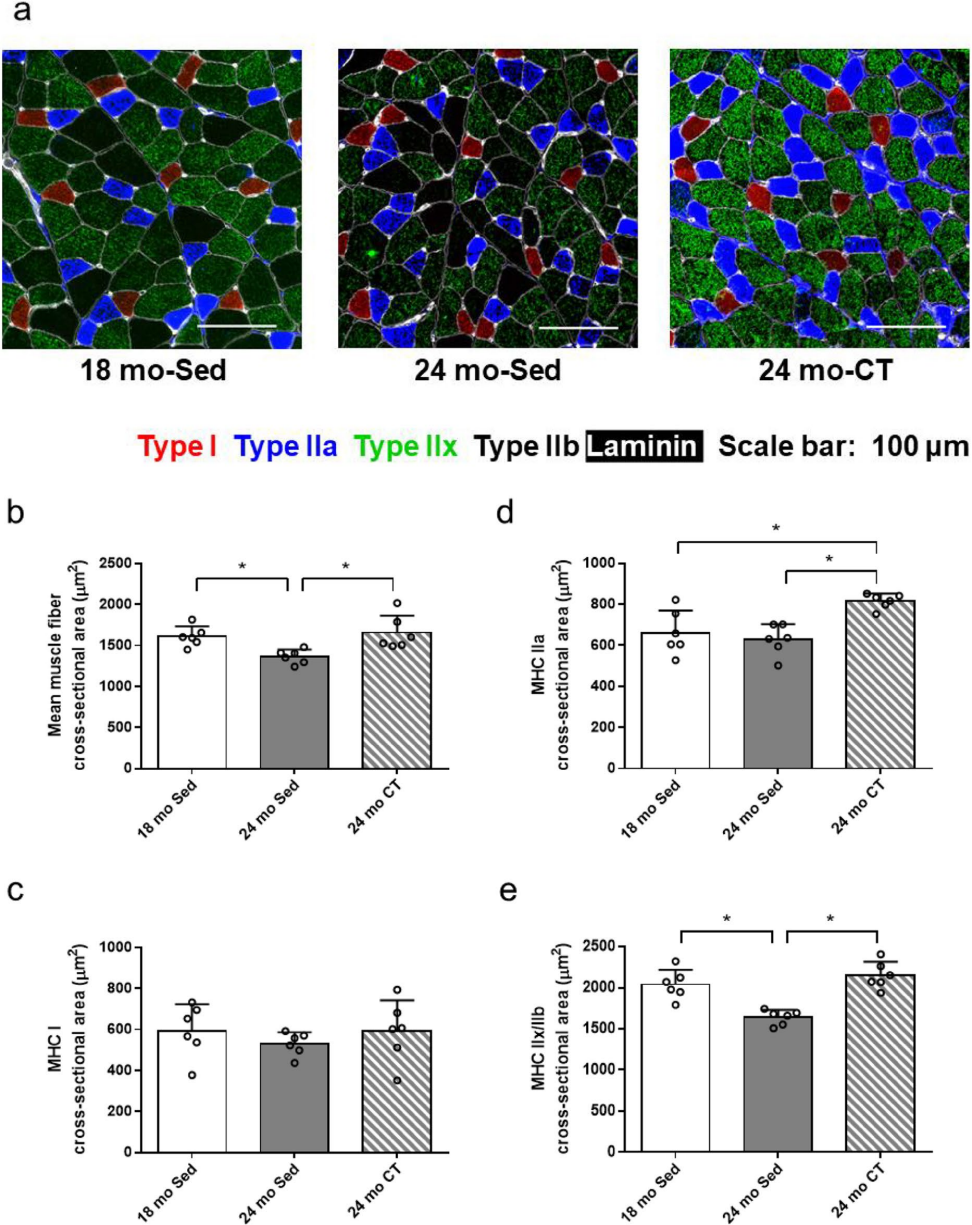
Effects of climbing training on the cross-sectional area of flexor hallucis longus (FHL) muscle. **a** Representative immunohistochemical staining of MHC. The reactivities of the major fiber types, including type I (red), type IIa (blue), and type IIx (green), are shown for FHL muscles. **b**–**e** The cross-sectional area of FHL muscle. **b** Mean muscle fiber cross-sectional area, **c **MHC I, **d** MHC IIa, **e** MHC IIx/IIb. Values are presented as the mean ± SD (*n* = 6); *: *p* < 0.05, Tukey post hoc test, scale bar: 100 μm. MHC, myosin heavy chain; 18 mo Sed, 18-month-old sedentary; 24 mo Sed, 24-month-old sedentary; 24 mo CT, 24-month-old climbing training; SD, standard deviation

The body weight was not significantly different among the study groups (18 mo-Sed group: 217.40 ± 27.35 g, 24 mo-Sed group: 235.17 ± 19.26 g, and 24 mo-CT group: 223.60 ± 14.87 g) and between pre- and post-training (22 mo-Sed group: 239.04 ± 19.04 g, and 22 mo-CT group: 241.94 ± 15.32 g). The FHL muscle mass was not significantly different between 18 mo-Sed group and 24 mo-Sed group; however, the FHL muscle mass was increased in the 24 mo-CT group, compared with those in the 24 mo-Sed group and 18 mo-Sed group (*p* < 0.001) (Fig. 1). On the CSA of FHL muscle (Fig. 2a), rats in the 24 mo-Sed group showed significant decreases (*p* = 0.008) in the mean muscle fiber CSA and type IIx/IIb muscle fiber CSA of FHL by 84.7 and 80.4%, respectively, compared to those in the 18 mo-Sed group. When compared to the 24 mo-Sed group, the mean muscle fiber CSA and type IIx/IIb muscle fiber CSA of FHL were significantly higher in the 24 mo-CT group. Furthermore, no significant difference was found between the 24 mo-CT group and the 18 mo-Sed group (Fig. 2b, e). Among all groups, the type I fiber CSA (Fig. 2c) was not significantly different for all treatments. The type IIa fiber CSA was increased in 24 mo-CT group, compared to those in the 24 mo-Sed and the 18 mo-Sed groups, although the increase in CSA was significantly different between the 24 mo-Sed group and 18 mo-Sed group (Fig. 2d). On the CSA related to the muscle mass, the mean muscle fiber CSA and type IIx/IIb muscle fiber CSA of FHL in the 24 mo-Sed group were significantly reduced compared with those in the 18 mo-Sed group. The mean muscle fiber CSA and type IIx/IIb muscle fiber CSA of FHL were not significantly different between the 24 mo-CT group and 18 mo-Sed group (Supplementary Fig. 1 a, d). The type I and IIa muscle fiber CSA of FHL showed no significant difference among all groups (Supplementary Fig. 1 b, c).

### Resistance training reduced senescent cell accumulation in skeletal muscles of aging rats

**Fig. 3:**
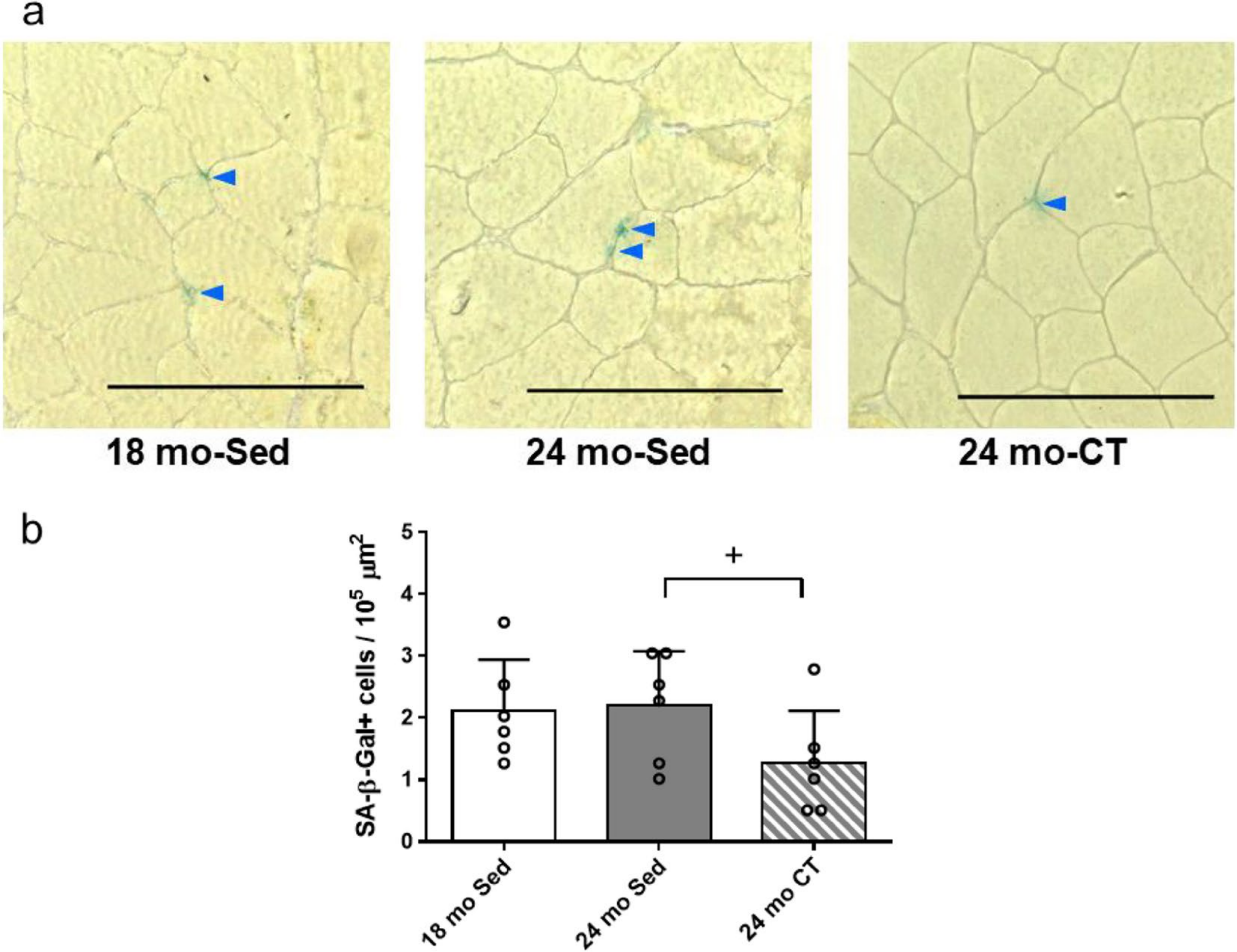
Effects of climbing training on the number of senescence-associated β-galactosidase (SA β-Gal)^+^ cell in flexor hallucis longus (FHL) muscle. **a** The representative immunohistochemical staining of SA β-Gal^+^ cells, and **b** the number of SA β-Gal^+^ cells in FHL muscle. Values are presented as the mean ± SD (*n* = 6); + : *p* < 0.05, unpaired Student’s *t* tests, scale bar: 100 μm. 18 mo Sed, 18-month-old sedentary; 24 mo Sed, 24-month-old sedentary; 24 mo CT, 24-month-old climbing training; SD, standard deviation

**Fig. 4:**
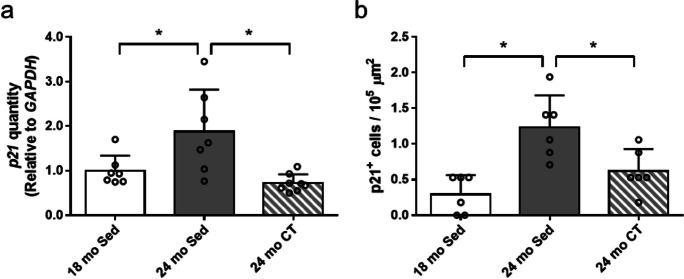
Effects of climbing training on *p21* gene expression and the number of p21^+^ cells in flexor hallucis longus (FHL) muscle. **a** The *p21* gene expression, and **b** the number of p21^+^ cells in FHL muscle. Values are presented as the mean ± SD (p21 gene expression (*n* = 7–8), the number of p21^+^ cell (*n* = 6)); *: *p* < 0.05, Tukey post hoc test. 18 mo Sed, 18-month-old sedentary; 24 mo Sed, 24-month-old sedentary; 24 mo CT, 24-month-old climbing training; SD, standard deviation

The senescent cells were confirmed by using SA β-Gal staining, q-PCR and IHC of p21. The proportion of SA β-Gal^+^ cells was not significantly different between 24 mo-Sed group and 18 mo-Sed group (Fig. 3a, b), but dramatically increased in sedentary 28–32 mo female rats (*n* = 5) (Supplementary Fig. 2). Particularly, SA β-Gal^+^ cells were significantly reduced in 24 mo-CT group compared with that in the 24 mo-Sed group (*p* = 0.045) (Fig. 3b). The p21 gene expression was highest in 24 mo-Sed group when compared to 18 mo-Sed group and 24 mo-CT group (*p* = 0.003) (Fig. 4a). Furthermore, p21^+^ cells were significantly increased in 24 mo-Sed group, and significantly decreased in 24 mo-CT group (Fig. 4b).

### Senescent FAPs were decreased in skeletal muscles of aging rats following resistance training

**Fig. 5:**
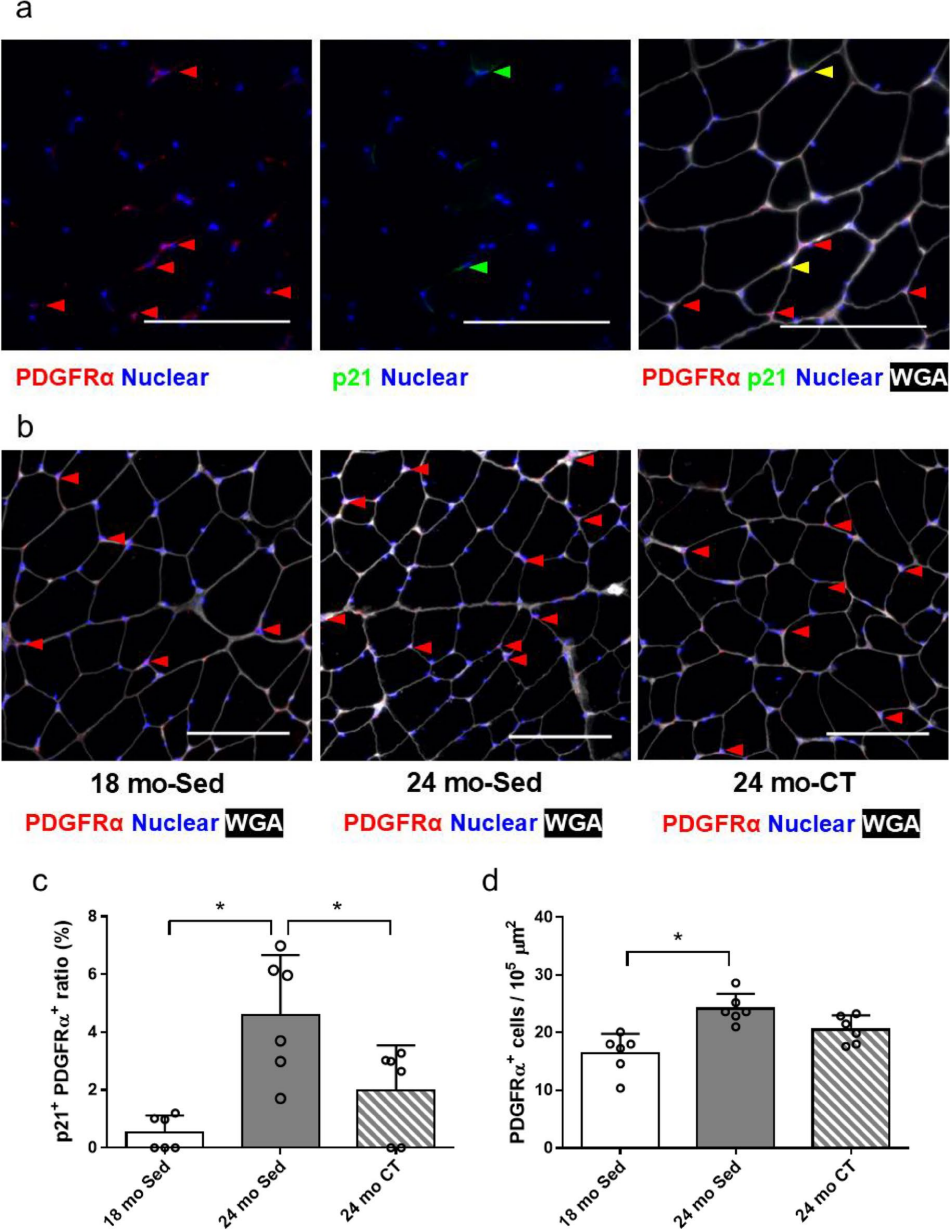
Effects of climbing training on the number of p21^+^ and PDGFRα^+^ cells in flexor hallucis longus (FHL) muscle. **a** The representative immunohistochemical staining of the number of p21^+^ and PDGFRα^+^ cells, the senescent fibro-adipogenic progenitors (FAPs) with PDGFRα^+^ (red) and p21^+^ (green), nuclear (blue), and wheat germ agglutinin (WGA) (white). The PDGFRα^+^ cells are indicated red arrowheads. The p21^+^ cells are indicated green arrowheads. The PDGFRα^+^ p21^+^ cells are indicated yellow arrowheads. **b** The representative immunohistochemical staining of the number of PDGFRα^+^ cell, the FAPs with PDGFRα^+^ (red) and nuclear (blue), and WGA (white). The PDGFRα^+^ cells are indicated red arrowheads. **c** Number of p21^+^ and PDGFRα^+^ cells and (**d)** the number of PDGFRα^+^ cells. Values are presented as the mean ± SD (*n* = 6); *: *p* < 0.05, Tukey post hoc test, scale bar: 100 μm. 18 mo Sed, 18-month-old sedentary; 24 mo Sed, 24-month-old sedentary; 24 mo CT, 24-month-old climbing training; SD, standard deviation

**Fig. 6:**
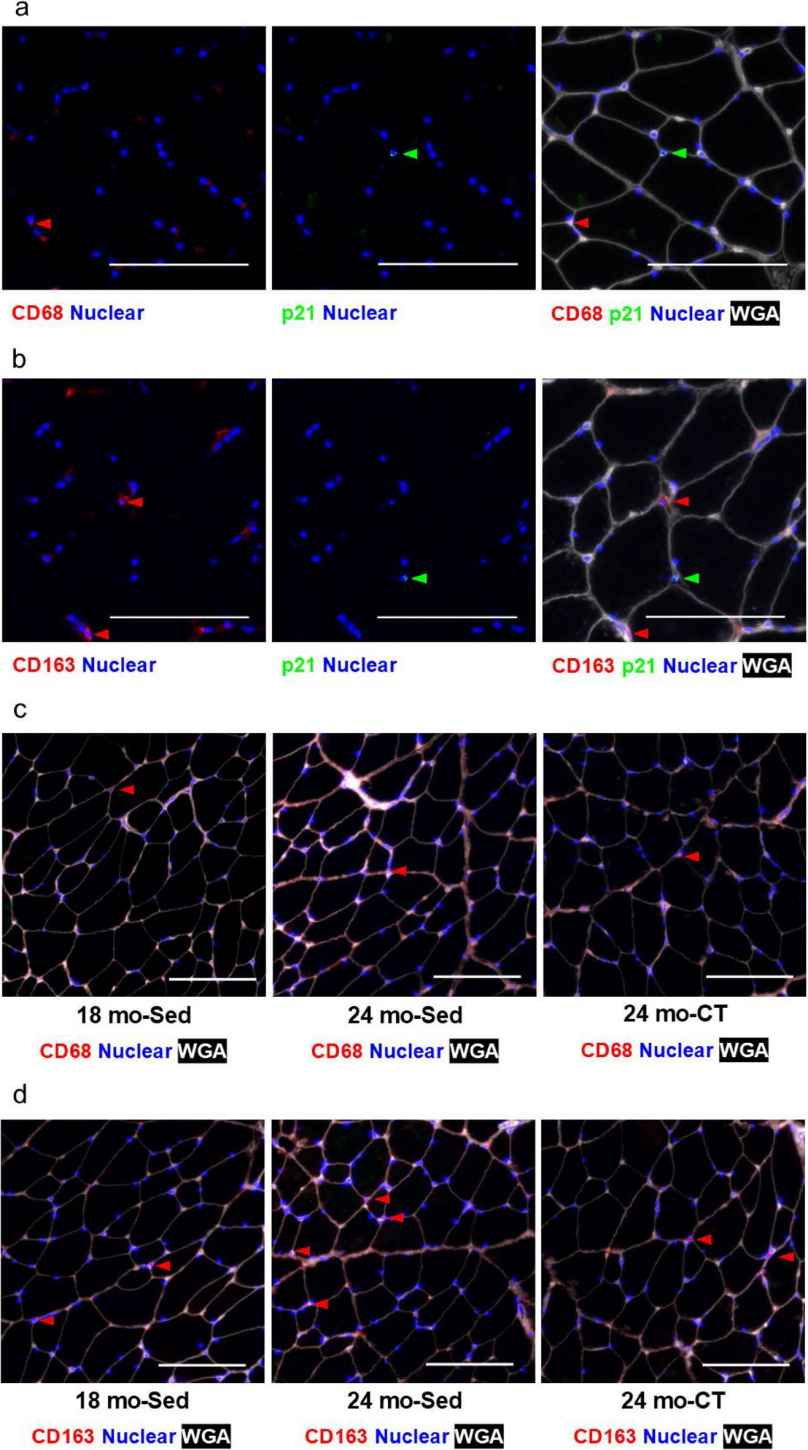

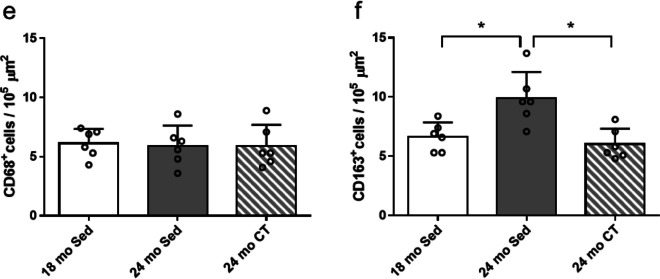
Effects of climbing training on the number of CD68^+^ and CD163^+^ cells in the flexor hallucis longus (FHL) muscle. **a** The representative immunohistochemical staining of the number of p21^+^ and CD68^+^ cells, the pan macrophage with CD68^+^ (red), nuclear (blue), and wheat germ agglutinin (WGA) (white). The senescent cells with p21^+^ (green). The CD68^+^ cells are indicated red arrowheads. The p21^+^ cells are indicated green arrowheads. **b** The representative immunohistochemical staining of the number of p21^+^ and CD163^+^ cells, the M2 macrophage with CD163^+^ (red), nuclear (blue), and WGA (white). The senescent cells with p21^+^ (green). The CD163^+^ cells are indicated red arrowheads. The p21^+^ cells are indicated green arrowheads. **c** The representative immunohistochemical staining of the number of CD68^+^ cells, the pan macrophage with CD68^+^ (red), nuclear (blue), and WGA (white). The CD68^+^ cells are indicated red arrowheads. **d** The representative immunohistochemical staining of the number of CD163^+^ cell, the M2 macrophage with CD163^+^ (red), nuclear (blue), and WGA (white). The CD163^+^ cells are indicated red arrowheads. **e** The number of CD68^+^ cells and (f) CD163^+^ cells. Values are presented as the mean ± SD (*n* = 6); *: *p* < 0.05, Tukey post hoc test, scale bar: 100 μm. 18 mo Sed, 18-month-old sedentary; 24 mo Sed, 24-month-old sedentary; 24 mo CT, 24-month-old climbing training; SD, standard deviation

The cell type of the senescent cells in skeletal muscle was examined by IHC of PDGFR-α (FAPs), CD68 (pan macrophage), and CD163 (M2 macrophage) (Fig. 5a and 6a, b). FAPs (PDGFR-α^+^ cells) increased significantly in the 24 mo-Sed group, compared to the 18 mo-Sed group (*p* = 0.001) (Fig. 5b, d). The substantial proportion of senescent cells (p21^+^ cells) were also FAPs (PDGFR-α^+^ cells) (Fig. 5a). On the other hand, almost all p21^+^ cells were either pan macrophages (CD68^+^ cells) or M2 macrophages (CD163^+^ cells) in aging skeletal muscles (Fig. 6a, b). The 24 mo-Sed group showed considerably higher levels of senescent FAPs (p21^+^ and PDGFR-α^+^ cells) compared to the 18 mo-Sed group (*p* = 0.001). Furthermore, the senescent FAPs were significantly reduced in 24 mo-CT group, as compared to the 24 mo-Sed group (Fig. 5c) (*p* = 0.001).

### Resistance training improved the skewed macrophage polarization in aging skeletal muscles

The effect of resistance training on macrophage polarization in skeletal muscles was confirmed. The pan macrophages (CD68^+^ cells) were not significantly different among the study groups (Fig. 6c, e). On the contrary, M2 macrophages (CD163^+^ cells) increased significantly in the 24 mo-Sed group compared with that in the 18 mo-Sed group (*p* = 0.002). Moreover, M2 macrophages were significantly reduced in the 24 mo-CT group compared with those in the 24 mo-Sed group (Fig. 6d, f) (*p* = 0.002).

### Resistance training suppressed SASP in skeletal muscles of aging rats

**Fig. 7:**
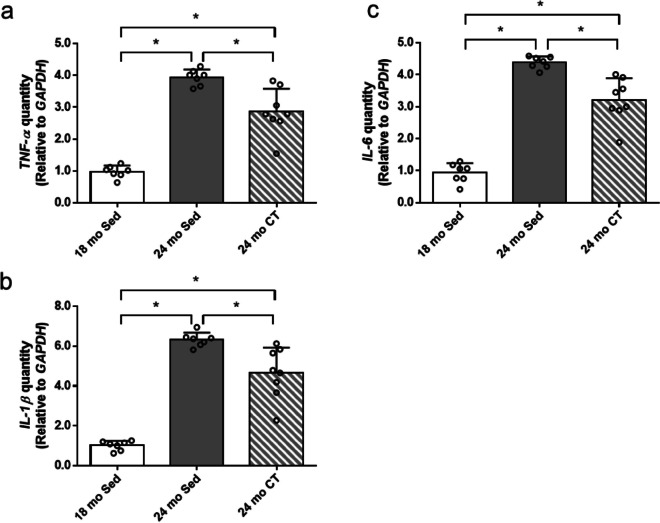
Effects of climbing training on senescence-associated secretory phenotype (SASP) in flexor hallucis longus (FHL) muscle. The gene expression of (a) *TNF-α*, **b** *IL-1β* and (**c**) *IL-6*. Values are presented as the mean ± SD (*n* = 7–8); *: *p* < 0.05, Tukey post hoc test. 18 mo Sed, 18-month-old sedentary; 24 mo Sed, 24-month-old sedentary; 24 mo CT, 24-month-old climbing training; TNF, tumor necrosis factor; IL, interleukin; SD, standard deviation

To identify the effect of resistance training on SASP in skeletal muscle, the gene expressions of SASP-related cytokines (TNF-α, IL-1β, and IL-6) in skeletal muscle were examined (Fig. 7). Gene expression of *SASP* (*TNF-α*, *IL-1β*, and *IL-6*) were significantly upregulated in 24 mo-Sed and CT groups compared with that in the 18 mo-Sed group (*p* < 0.001). Notably, the expression of *TNF-α*, *IL-1β*, and *IL-6* were significantly reduced in aging rats following resistance training, compared with those in the 24 mo-Sed group (Fig. 7a, b, c) (*p* < 0.001).

### Killer T cell-specific marker was increased in skeletal muscles of aging rats following resistance training

**Fig. 8:**
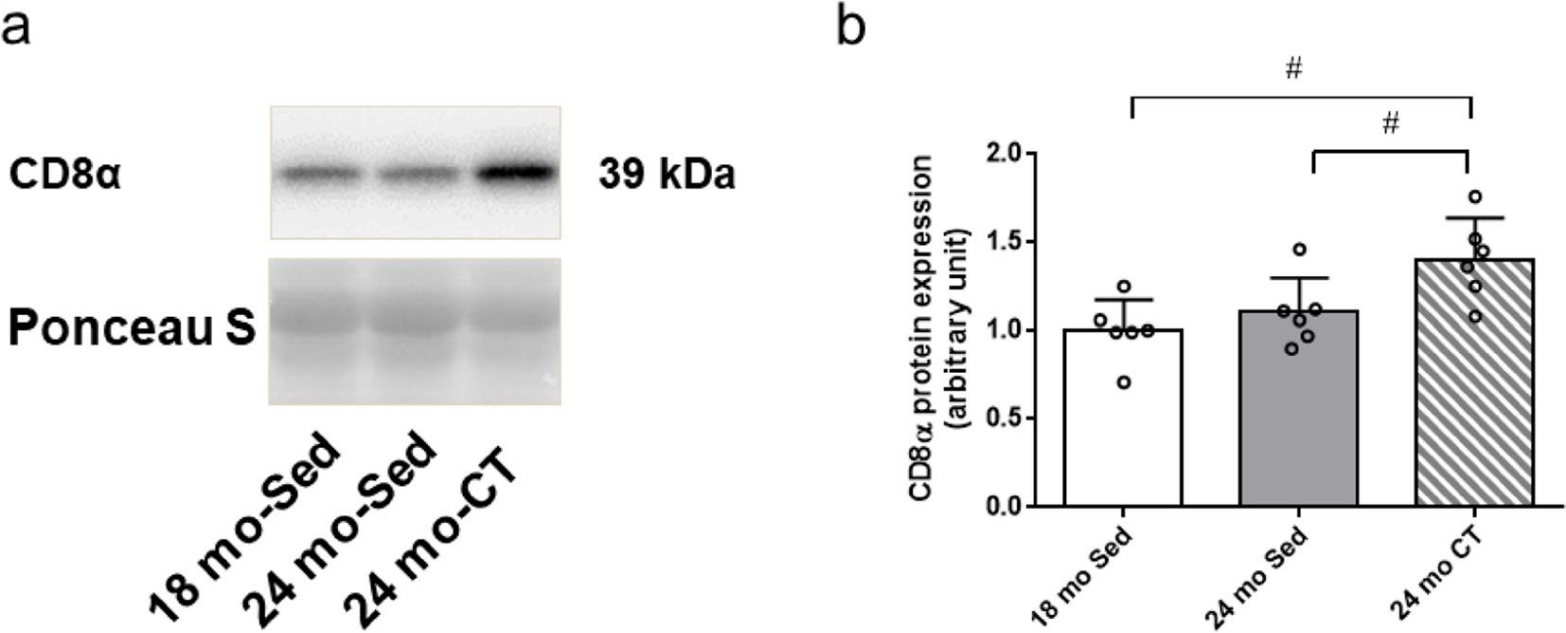
Effects of climbing training on CD8α in flexor hallucis longus (FHL) muscle (**a**) The expressions of CD8α were determined by western blotting, **b** protein level of CD8α. Values are presented as the mean ± SD (*n* = 6); #: *p* < 0.05, Fisher’s LSD test. 18 mo-Sed, 18-month-old sedentary; 24 mo-Sed, 24-month-old sedentary; 24 mo-CT, 24-month-old climbing training; SD, standard deviation

The CD8^+^ T cells play a critical role in recognizing and eliminating senescent cells [23]. Thus, the killer T cell-specific marker CD8α in skeletal muscles was analyzed. The CD8α protein was not significantly different between the 24 mo-Sed and 18 mo-Sed groups. Interestingly, CD8α protein levels were significantly higher in the 24 mo-CT group compared to the 24 mo-Sed and 18 mo-Sed groups (*p* = 0.021) (Fig. 8a, b).

## Discussion

In the present study, the effects of resistance training on cellular senescence in aging skeletal muscles were investigated. The experimental findings showed that resistance training alleviated aging-associated skeletal muscle atrophy while suppressing M2 polarization of macrophages in aging skeletal muscles. Furthermore, resistance training reduced the accumulation of senescent cells and SASP-associated proinflammatory molecules. Resistance training, additionally, raised the CD8α protein levels, reflecting the presence of killer T cell in skeletal muscles.

The mean muscle fiber CSA of FHL was markedly reduced in sedentary 24-month-old rats when compared with sedentary 18-month-old rats. Especially, the fast twitch type IIx/IIb muscle fiber CSA was significantly decreased in sedentary 24-month-old female F344 rats in the present study. These findings are consistent with other studies on aged animal models [24, 25]. Thus, the aged female F344 rats can be considered a suitable animal model for investigating sarcopenia. Resistance training is known to improve skeletal muscle mass in aging [19]. The mean muscle fiber CSA and type IIx/IIb muscle fiber CSA of FHL were increased in the aged female F344 rats following resistance training. This indicates that resistance training can attenuate aging-associated skeletal muscle atrophy in aged female F344 rats.

In the present study, the SA β-Gal^+^ cells were not significantly elevated in aging animals, though p21^+^ cells were markedly increased in 24 mo-Sed female rat skeletal muscles. Dungan et al. [26] indicated that skeletal muscles contain very few SA β-Gal^+^ cells and p21^+^ cells, with no difference between adult (5–6 months) and old (23–24 months) mice. Moreover, SA β-Gal^+^ cells were significantly increased in geriatric (28–32 months) female rat skeletal muscles in the present study. It suggested that SA β-Gal^+^ cell is a marker of cellular senescence in geriatric (late stage of aging), not old (early stage of aging) skeletal muscles. The p21 is significantly increased in skeletal muscles of old (24 months) mice, and thus considered to be a distinguishing factor of cellular senescence in skeletal muscles [27]. Elevated p21 was shown to induce skeletal muscle SASP, atrophy, fibrosis, and decline in physical function in mouse model of p21 overexpression [28]. Most p21^+^ cells were located outside muscle fibers, and presented cytoplasmic p21 in the present study. These experimental results were in agreement with a previous study [26]. Renty et al. [29] demonstrated that the cytoplasmic localization of p21 prevented DNA damage-induced apoptosis. The cytoplasmic localization of p21 could be associated with senescent cell antiapoptotic pathways (SCAPs) in senescent cells. Taken together, the experimental results of the present study demonstrated that there is an accumulation of senescent cells in skeletal muscles in sedentary 24-month-old female rats.

Our experimental results indicated that the major senescent cells (p21^+^ cells) are FAPs (PDGFR-α^+^ cells) in aging skeletal muscles. Zhang et al. [27] demonstrated that FAPs exhibit high cellular senescence among mitotically competent monocular cells (endothelial cells, macrophages, satellite cells, and FAPs) in aging skeletal muscle. The senolytic treatment could reduce both SA β-Gal^+^ cells and PDGFR-α^+^ cells in progeria-aged skeletal muscle [16]. This suggests that the major senescent cells in skeletal muscles are the senescent FAPs. We also found the total FAPs to be elevated in skeletal muscle of aging animals, which can be compared to the increase in progeria-aged skeletal muscle [16]. On the contrary, total FAPs were not significantly increased in skeletal muscle following resistance training. Additionally, the senescent FAPs (p21^+^ and PDGFR-α^+^ cells) were markedly reduced in skeletal muscles following resistance training. These findings demonstrate that resistance training exhibits similar effects as senolytic treatment on senescent FAPs in skeletal muscles.

M2 macrophage is a cellular source of transforming growth factor beta (TGFβ), which prevents the apoptosis of FAPs [14]. Furthermore, the M2 macrophage-derived TGFβ, fibroblast growth factor (FGF), and PDGF support fibroblast growth and activation, and induce myofibroblast-like or inflammatory phenotypes [30]. Increasing M2 macrophages skew macrophage polarization and contribute to fibrosis in aging skeletal muscles [31]. Conversely, Zhang et al. [32] showed that senescent skeletal muscle FAPs recruit macrophages and promote M2 polarization of macrophages in vitro. They suggested that FAPs contribute to maintain macrophage polarization and muscle homeostasis. The results in the present study showed that resistance training suppressed M2 polarization of macrophages in aging skeletal muscles. The decrease in M2 polarization of macrophages in aging skeletal muscles following resistance training might be associated with resistance training-suppressed accumulation of senescent FAPs.

The SASP is produced by accumulation of senescent cells, and have been implicated as a factor in development of sarcopenia [10]. The gene expressions of the *SASP* (*TNF-α*, *IL-1β*, and *IL-6*) were observed to be higher in skeletal muscle in old mice in our previous study [33]. Additionally, *SASP* gene is significantly upregulated in sedentary aged female rat skeletal muscles, and this increase in SASP was attenuated by resistance training. Elimination of senescent FAPs by the senolytic treatment was shown to reduce SASP in vitro [16]. Our results suggest that resistance training attenuated SASP via reduction of senescent cell accumulation in aged female rat skeletal muscles.

The CD8^+^ T cells critically contribute to recognition and elimination of senescent cells [34, 35]. CD8^+^ T cells restrict senescent cell accumulation, which improves immunosurveillance [35]. The CD8α protein did not significantly increase with aging in the present study. Moreover, aging did not significantly increase T cells (CD45^+^CD3e^+^ cells) in old mice skeletal muscles in our previous study [33]. Interestingly, resistance training increased CD8α protein in aging female rat skeletal muscles. The immune checkpoint protein programmed cell death 1 that can cause escape from immunosurveillance was shown to be significantly lowered in circulating CD3^+^ T cells in older adults following 12-week structured exercise program [36]. Thus, resistance training might improve immunosurveillance by CD8^+^ T cells in the present study. In contrast, the elevated level of CD8^+^ T cells might contribute to higher TNF-α, IL-1β, and IL-6 expression in skeletal muscles of aging female rats following resistance training. Taken together, the present experimental results suggest that resistance training can suppress senescent cell accumulation via CD8^+^ T cells in aging female rat skeletal muscles.

## Conclusion

Resistance training suppressed accumulation of senescent FAPs and SASP could via killer T cells in aging skeletal muscles. These results provide new insights into the mechanisms of improvement of aging-related skeletal muscle atrophy by resistance training, and a potential treatment option for sarcopenia. Although resistance training might influence immunosurveillance of T cells, the molecular mechanisms remain unclear, which needs to be clarified by single cell and bulk RNA-sequencing, and cellular senescence-related signaling analysis in the future studies.

## Supplementary Information

Below is the link to the electronic supplementary material.

Supplementary file1 (PDF 143 KB)

## Data Availability

The data that support the findings of this study are available from the corresponding author, Prof. Machida, upon reasonable request.
