# Supplementary figures and images for: Resistance training suppresses accumulation of senescent fibro-adipogenic progenitors and senescence-associated secretory phenotype in aging rat skeletal muscle
Source: GeroScience. 2024 Sep 19;47(2):1669–83. doi: 10.1007/s11357-024-01338-2 (PMC11979060; doi:10.1007/s11357-024-01338-2)

Supplemental Fig. 1

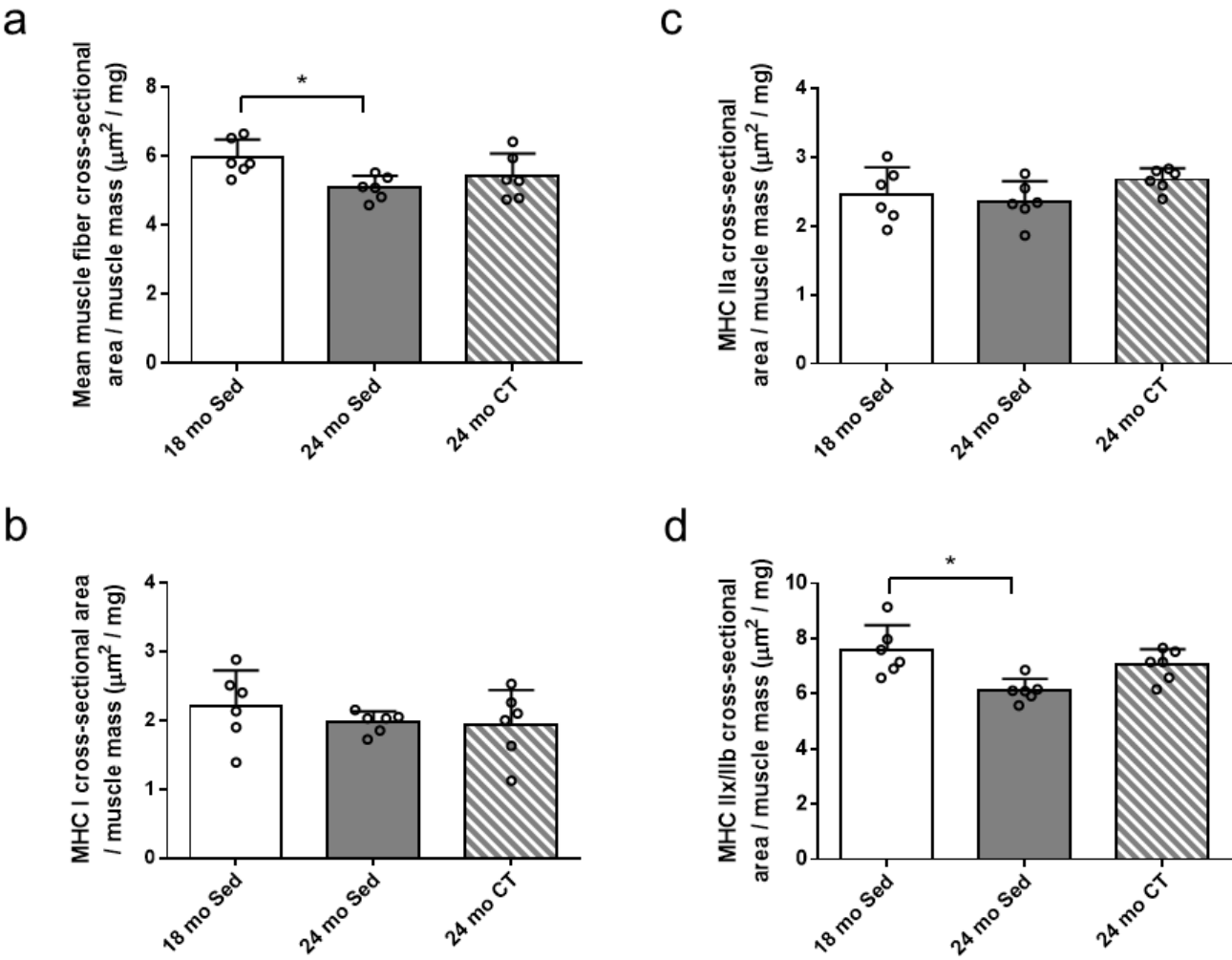

Supplemental Fig. 2

a

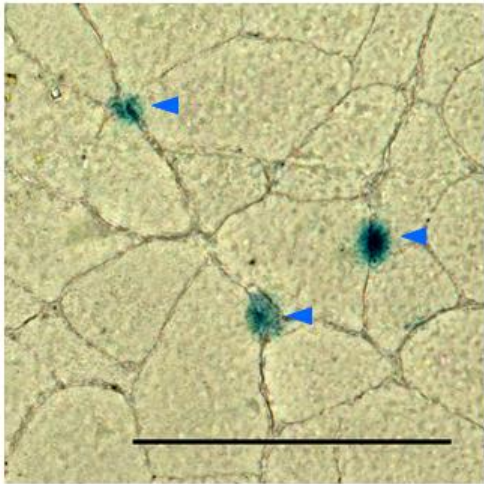

28 mo-Sed

b

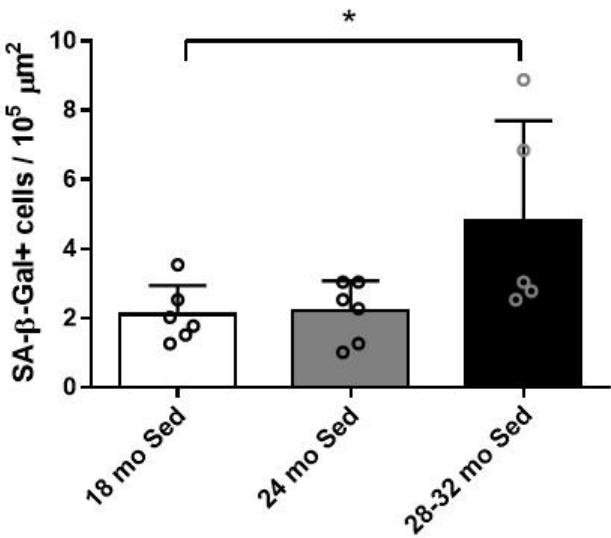

Supplement: Supplementary file 1 — Supplementary file1 (PDF 143 KB) [file 11357_2024_1338_MOESM1_ESM.pdf]
